# COVID-19-related anxiety and sense of coherence associated with psychological distress among rural residents in Japan during the pandemic late phase

**DOI:** 10.1265/ehpm.25-00457

**Published:** 2026-05-16

**Authors:** Kaori Taniguchi, Atsuko Sadakane, Shunsuke Yamashina, Shinya Matsumoto, Kiwamu Nagoshi

**Affiliations:** Department of Environmental Medicine and Public Health, Faculty of Medicine, Shimane University, 89-1 Enya-Cho, Izumo, Shimane 693-8501, Japan

**Keywords:** COVID-19, Sense of coherence, Psychological distress, Rural health, Japan, Economic anxiety, Mask-wearing

## Abstract

**Background:**

The coronavirus disease 2019 (COVID-19) pandemic has imposed significant psychological stress worldwide. Although many studies have examined the mental health impacts during the pandemic’s early phase, evidence from the late phase remains limited. This study investigated the associations between COVID-19-related anxiety and sense of coherence (SOC) with psychological distress among rural residents during the pandemic late phase in Japan.

**Methods:**

This community-based cross-sectional study was conducted from July 2022 to March 2023, the late phase of the COVID-19 pandemic. The participants were residents of rural communities in the Shimane Prefecture, Japan. They were recruited through a health check-up program arranged by a cooperative organization for farmers. Psychological distress was assessed using the Kessler 6-item Psychological Distress Scale (K6); COVID-19-related anxiety and SOC were assessed using self-administered questionnaires. Multivariable logistic regression analyses were performed to examine the associations between pandemic-related anxiety, SOC, and psychological distress after adjusting for age, sex, subjective health status, and communicative health literacy.

**Results:**

Of the 1,310 individuals who underwent health checkups, 743 agreed to participate in the study, and 664 who completed all questionnaires were included in the final analyses. Psychological distress (K6 ≥ 5) was observed in 29.2% of participants and was more prevalent among younger adults. Fear of infection (OR = 1.32, 95% confidence interval [CI]: 1.05–1.68), stress related to mask wearing (OR = 1.35, 95% CI: 1.10–1.68), and concern about reduced income (OR = 1.32, 95% CI: 1.08–1.61) were associated with higher odds of psychological distress. Higher SOC was inversely associated with psychological distress.

**Conclusions:**

During the late phase of the COVID-19 pandemic, several COVID-19-related anxiety factors, particularly fear of infection, stress related to mask wearing, and concern about reduced income, were associated with psychological distress among rural residents. Higher SOC was associated with lower psychological distress, suggesting that strengthening individual and community resilience may be important for mitigating the mental health impact of prolonged pandemic conditions in rural settings.

## Background

The coronavirus disease 2019 (COVID-19) pandemic, from January 2020 to May 2023, has affected the physical, mental, and social well-being of individuals for a prolonged period. In the early phases of the pandemic, public health and social preventative measures, including stay-at-home orders, restrictions on economic activities, and physical distancing, were rapidly implemented in many countries following the World Health Organization recommendations and national policy guidance [1–3]. In Japan, repeated state of emergency declarations and quasi-emergency measures issued between 2020 and 2022 under the Act on Special Measures for Pandemic Influenza and New Infectious Diseases [4, 5] have substantially affected daily life, work environments, and social relationships. These changes likely contributed to increased psychological stress by heightening social isolation, anxiety, and financial hardship. Numerous studies have reported an elevated prevalence of anxiety and depressive symptoms across countries and socio-demographic groups during this period [6, 7].

The prolonged nature of the pandemic has created a chronically stressful environment, raising concerns about the psychological health consequences, such as anxiety and depression [8, 9]. Under such difficult circumstances, individual stress-coping capacities play an essential role in maintaining good mental health. In this context, sense of coherence (SOC), which is a core concept in Antonovsky’s salutogenic model [10], has garnered much attention as a psychological resource that enables individuals to maintain their health under sustained stress. SOC reflects the extent to which individuals perceive life events as comprehensible, manageable, and meaningful components [11]. As SOC emphasizes mechanisms that allow individuals to mobilize internal and external resources for well-being, it is considered highly relevant during prolonged public health crises [10, 12].

Given that the pandemic has created sustained and multifaceted stress across diverse populations, SOC may have played an important protective role by helping individuals reframe stressors, access available resources, and maintain a sense of purpose under those challenging circumstances. Studies conducted across various cultural and social contexts demonstrated that SOC buffered adverse psychological impacts from the COVID-19 pandemic, reduced anxiety and distress, and contributed to better mental health outcomes [13–16].

Despite these important contributions, several critical gaps remain in the literature. Most previous studies were conducted during the early phase of the pandemic (2020–2021) and primarily focused on populations whose daily lives were substantially disrupted, such as metropolitan residents [6, 7], healthcare workers [17, 18], university students [19, 20], and individuals transitioning to remote work [21]. Although these groups experienced considerable and abrupt lifestyle changes, less attention has been paid to rural regions, characterized by low population density and aging individuals, where the nature and intensity of pandemic-related stressors may have substantially differed. However, research in rural settings, particularly in Japan and other Asian countries, is scarce. Moreover, a limited number of studies have examined the impact of COVID-19 on mental health during the late phase of the pandemic. Prolonged exposure to stressors, behavioral fatigue, and shifting risk perceptions may have altered patterns of stress and coping during this late phase.

To address these gaps, we conducted a community-based study during the late phase of the COVID-19 pandemic in the Shimane Prefecture, Japan. Shimane is a representative rural region characterized by its lengthy distance from any major urban area and a small population (641,396 people as of October 1, 2024; approximately 0.5% of the total population in Japan), with a high percentage of older adults (35% vs. 29% in Shimane and the national average, respectively) [22] and agricultural workers (6.6% vs. 2.9% in Shimane and the national average, respectively) [23, 24]. Therefore, we aimed to investigate the relationship between COVID-19-related anxiety, SOC, and psychological distress among rural residents in the late phase of the COVID-19 pandemic. This study may provide important information about community resilience during a persistent pandemic.

## Methods

### Study design and setting

This cross-sectional study was conducted in rural communities in Shimane between July 2022 and March 2023, approximately 2.5 years after the COVID-19 pandemic onset. The seventh and eighth waves of infections emerged during the study period, although most pandemic-related restrictions in Japan had eased.

### Participants

The participants were residents of six communities in the Shimane Prefecture, Japan, and members of the Japan Agricultural Cooperatives (JA), a cooperative organization that supports farmers and rural communities and provides a wide range of services, including agricultural support, financial services, and health-related programs. The participants were recruited from among those who underwent comprehensive annual health checkups during the study period from July 15, 2022, to March 31, 2023. Of the 1,310 individuals who underwent health checkups during the study period, 743 agreed to participate in the study and provided written informed consent. After excluding individuals with missing data, 664 participants were included in the final analyses.

### Data sources and measurements

All the data were obtained from the self-administered questionnaires. The participants were instructed to complete the questionnaires at home before their health checkups and submit them in sealed envelopes on the day of the checkup. The questionnaires included one regarding COVID-19-related anxiety, the Kessler 6-item Psychological Distress Scale (K6), and the 13-item Sense of Coherence Scale (SOC-13); and demographic and lifestyle factors were also obtained. Participant data were provided by the JA in an anonymized form, with all personal identifiers removed.

### Variables and definitions

#### COVID-19-related anxiety

To assess pandemic-related anxiety, a questionnaire consisting of eight items was developed with reference to a national survey on mental health during the COVID-19 pandemic conducted by the Ministry of Health, Labour and Welfare of Japan in 2021 [25]. Questions inquired about anxiety due to (1) fear of infection, (2) fear of discrimination if infected, (3) stress about wearing a mask, (4) restriction of leisure or travel, (5) restriction of occasions to meet family or friends, (6) difficulty dining out, (7) concern about reduced income, and (8) difficulty accessing health care. Each item was rated on a 5-point Likert scale (1 = strongly disagree, 2 = disagree, 3 = neither agree nor disagree, 4 = agree, 5 = strongly agree). The scale demonstrated good internal consistency in this study (Cronbach’s α = 0.80).

#### Psychological distress

The Kessler Psychological Distress Scale (K6) has been widely used as a reliable screening tool to assess population-level mental health status in various populations, including Japanese people [26, 27]. The K6 comprises six items rated on a 5-point Likert scale, with response options ranging from 0 to 4 (0 = none of the time, 1 = a little of the time, 2 = some of the time, 3 = most of the time, and 4 = all the time). The total score ranges from 0 to 24, with higher scores indicating greater psychological distress. Consistent with previous population-based studies, we defined psychological distress as K6 ≥ 5, a threshold shown to have acceptable sensitivity and specificity for detecting moderate mental distress in community settings [28]. In this study, the internal consistency of the K6 was excellent (Cronbach’s α = 0.87). The “alpha if item deleted” values ranged from 0.83 to 0.96, indicating that all items contributed positively to the overall reliability of the scale.

#### SOC

SOC was assessed using the Japanese version of the 13-item SOC-13 [29]. Each item is rated on a 7-point Likert scale, yielding a total score ranging from 13 to 91, with higher scores indicating stronger SOC. The internal consistency of the SOC-13 scale was high (Cronbach’s α = 0.86). The “alpha if item deleted” values ranged from 0.84 to 0.86, indicating that all items contributed positively to the reliability of the scale.

#### Other variables

Age, sex, farming frequency, underlying diseases, smoking status, alcohol consumption, sleep condition, and exercise habit information was collected using the questionnaire. Lifestyle variables (smoking status, alcohol consumption, sleep conditions, and exercise habits) were assessed only among participants aged <75 years (n = 590) because the health checkup questionnaire differed between those aged ≥75 and <75 years. In addition, subjective health perception was obtained using a single item rated on a 5-point scale (1 = very bad, 2 = bad, 3 = normal, 4 = good, and 5 = very good) [30]. Communicative health literacy was also obtained using a 5-item scale based on the framework proposed by Ishikawa and Kiuchi [31]. This scale evaluates an individual’s ability to obtain, understand, communicate, and apply health-related information in daily life. Each item is rated on a 5-point Likert scale (1 = strongly disagree, 2 = disagree, 3 = neither agree nor disagree, 4 = agree and 5 = strongly agree), with higher scores indicating better communicative health literacy. The internal consistency of the scale was acceptable (Cronbach’s α = 0.80).

### Statistical analyses

Statistical analyses were conducted to describe participant demographic and lifestyle characteristics and examine the factors associated with psychological distress. Categorical variables were summarized as frequencies and percentages, and continuous variables were presented as means and standard deviations. Group differences were assessed using the chi-square test for categorical variables, Student’s *t*-test for means of two groups, and one-way analysis of variance (ANOVA) for means of three or more groups. To investigate the associations between COVID-19-related anxiety and psychological distress, we conducted a series of logistic regression analyses using dichotomized K6 scores as the dependent variable. Although the COVID-19-related anxiety items were treated as individual indicators rather than as a composite scale, we checked variance inflation factors (VIF) by entering all items in a linear regression model: all VIFs were <10, indicating little evidence of substantial multicollinearity among these items. One of the eight COVID-19-related anxiety items was treated as a continuous variable and used as the primary independent variable. SOC was also included as an independent variable in the form of a continuous variable in all models. To facilitate the interpretation of the effect size, SOC was standardized, and the associations were estimated per standard deviation (SD) increase in the SOC score. We controlled for age and sex as potential confounding factors. In addition, subjective health status and communicative health literacy were controlled for based on previous knowledge [31, 32]. Odds ratios (ORs) and 95% confidence intervals (CIs) were reported. A two-sided *p* value < 0.05 was considered statistically significant.

Because lifestyle variables (smoking status, alcohol consumption, sleep conditions, and exercise habits) were available only for participants aged <75 years due to differences in the health check-up questionnaires, we conducted a sensitivity analysis restricted to this subgroup and additionally adjusted for these lifestyle factors to examine the robustness of the findings.

All analyses were performed using JMP Pro version 17.2.0 (SAS Institute Inc., Cary, NC, USA).

## Results

A total of 664 participants were included in the analyses, with a proportion of men of 481 (72.4%). The mean age was 60.2 years for men and 59.1 years for the women. The percentage of those with a K6 score above the cutoff of 5 was 29.2%. The mean SOC-13 score was 63.5 (standard deviation = 12.1). To assess potential bias due to missing data, we compared the characteristics of participants included in the final analysis (n = 664) with those excluded because of missing questionnaire responses (n = 79). The excluded participants were somewhat older (mean age 68.0 years, SD = 12.6) than those included in the analysis (mean age 59.9 years, SD = 14.8), whereas the sex distribution was similar between the two groups (74.7% vs.72.4% men).

Table 1 presents the proportions of participants classified as having K6 ≥ 5 and the mean SOC-13 scores according to demographic and lifestyle factors. The proportion of participants with K6 ≥ 5 did not differ significantly across age groups (p = 0.138); however, the prevalence tended to be higher among those aged 20–39 years and lower among those aged ≥70 years. The SOC-13 scores also differed significantly by age (p < 0.001), showing an increasing trend with advancing age. Neither the prevalence of K6 ≥ 5 nor SOC-13 scores differed significantly according to disease status, farming frequency, drinking frequency, smoking status, or exercise habits. However, the participants who reported sufficient sleep had significantly higher SOC-13 scores than those who reported insufficient sleep (65.0 vs. 60.0, p < 0.001).

**Table 1:** Psychological distress prevalence (K6 ≥ 5) and mean SOC-13 by demographics and lifestyles (n = 664)

**Variables**	**Categories**	**n**	**K6 ≥ 5**	p-value*	**SOC-13 Mean (SD)**	p-value*
**Sex**	Male	481	27.9%	0.212	64.1(11.6)	0.055
	Female	183	32.8%		62.1(13.1)	
**Age (years)**	20–29	31	38.7%	0.138	58.5(12.3)	<0.001
	30–39	47	42.6%		58.4(15.0)	
	40–49	103	28.2%		60.9(11.9)	
	50–59	62	33.9%		60.5(10.8)	
	60–69	203	30.1%		64.8(11.3)	
	70–79	202	23.3%		65.9(11.6)	
	≥80	16	25.0%		70.8(10.2)	
**Farming frequency**	Daily	413	21.8%	0.129	63.4(12.2)	0.218
	2–3 times/week	113	12.8%		64.8(11.9)	
	Once/week	60	26.7%		64.2(11.0)	
	Once/month	13	23.1%		62.0(13.7)	
	Not farmers	45	24.4%		60.1(11.3)	
**Underlying diseases**	Present (≥2 diseases)	274	29.6%	0.714	64.3(11.4)	<0.001
	Present	130	26.2%		64.8(12.4)	
	No diseases	224	31.3%		61.2(12.2)	
	Cured	36	25.0%		68.2(12.5)	
Smoking status**	Non-smokers	477	31.2%	0.178	62.5(11.9)	0.045
	Smokers	113	24.8%		65.0(11.3)	
Alcohol consumption**	Non-drinkers	244	32.0%	0.382	61.9(11.6)	0.095
	Occasionally	121	32.2%		62.8(12.1)	
	Daily	225	26.8%		64.3(12.0)	
Sleep condition**	Sufficient	357	22.7%	<0.001	65.0(11.7)	<0.001
	Insufficient	233	41.2%		60.0(11.6)	
Exercise habit**	Regular	141	28.4%	0.628	64.1(11.6)	0.221
	None	449	30.5%		62.7(11.9)	
**Self-rated health**	Very bad	13	69.2%	<0.001	57.2(10.5)	<0.001
	Bad	99	43.4%		58.8(11.1)	
	Normal	453	28.0%		63.3(11.8)	
	Good	83	15.7%		70.4(11.2)	
	Very good	16	12.5%		69.1(12.0)	
**Health literacy**	1st quartile (lowest)	162	26.5%	0.141	63.3(12.2)	0.151
	2nd quartile	185	27.0%		62.6(11.8)	
	3rd quartile	174	27.6%		65.3(12.7)	
	4th quartile (highest)	143	37.1%		62.8(11.4)	

*For K6 ≥ 5, p-values were calculated using the chi-square test.

For SOC-13 mean scores, p-values were calculated using Student’s t-test (two groups) or one-way analysis of variance (ANOVA) (three or more groups), as appropriate.

P-values are presented to three decimal places; values <0.001 are shown as <0.001.

**Lifestyle variables (smoking status, alcohol consumption, sleep condition, and exercise habit) were assessed only among participants aged <75 years (n = 590).

Table 2 shows the proportions of those with K6 ≥ 5 and SOC-13 scores according to their responses to each COVID-19-related stressor item. For most items, K6 ≥ 5 proportions increased as responses shifted from “strongly disagree” to “strongly agree.” In contrast, SOC-13 scores decreased as the responses shifted toward “strongly agree.” The participants who strongly agreed with stress-related statements, such as, “I am worried that I or my family might become infected,” “I might be discriminated against if infected,” “My work or income has decreased,” and “It has become difficult to receive medical services,” had higher percentages of K6 ≥ 5 and lower mean SOC-13 scores than those who disagreed.

**Table 2:** Psychological distress prevalence (K6 ≥ 5) and mean SOC-13 by COVID-19 stressor responses (n = 664)

	**n (%)**	**K6 ≥ 5**	**SOC Mean (SD)**
**Fear of infection**			

Strongly disagree	17(2.6)	17.7%	67.1(11.9)
Disagree	85(12.8)	18.8%	67.9(11.1)
Neither agree nor disagree	168(25.3)	21.4%	63.4(11.6)
Agree	318(47.9)	30.8%	63.7(11.9)
Strongly agree	76(11.5)	54.0%	57.3(12.3)

**Fear of discrimination if infected**			

Strongly disagree	85(12.8)	23.5%	67.0(12.6)
Disagree	309(46.5)	24.9%	64.8(11.8)
Neither agree nor disagree	166(25.0)	27.7%	62.0(11.4)
Agree	94(14.2)	48.9%	59.6(11.6)
Strongly agree	10(1.5)	50.0%	55.2(15.9)

**Stress about wearing a mask**			

Strongly disagree	38(5.7)	7.9%	69.6(11.6)
Disagree	59(8.9)	17.0%	66.2(12.3)
Neither agree nor disagree	85(12.8)	30.6%	61.9(10.5)
Agree	298(44.9)	27.2%	63.6(11.9)
Strongly agree	184(27.7)	40.2%	62.0(12.6)

**Restriction of leisure or travel**			

Strongly disagree	40(6.0)	17.5%	65.8(12.2)
Disagree	175(26.4)	23.4%	64.6(11.6)
Neither agree nor disagree	154(23.2)	29.9%	63.6(11.5)
Agree	230(34.7)	32.2%	62.9(11.9)
Strongly agree	65(9.8)	40.0%	61.2(14.9)

**Restriction of occasions to meet family or friends**			

Strongly disagree	59(8.9)	17.0%	65.8(12.2)
Disagree	231(34.8)	26.0%	64.9(11.5)
Neither agree nor disagree	146(22.0)	28.8%	62.8(11.3)
Agree	175(26.4)	36.0%	62.0(12.3)
Strongly agree	53(8.0)	35.9%	61.9(14.7)

**Difficulty dining out**			

Strongly disagree	49(7.4)	16.3%	66.3(12.1)
Disagree	199(30.0)	26.6%	65.0(11.7)
Neither agree nor disagree	151(22.7)	28.5%	64.8(11.8)
Agree	211(31.8)	32.7%	61.2(12.0)
Strongly agree	54(8.1)	38.9%	61.1(12.6)

**Concern about reduced income**			

Strongly disagree	54(8.1)	13.0%	67.0(12.6)
Disagree	187(28.2)	20.3%	66.2(10.8)
Neither agree nor disagree	220(33.1)	30.0%	62.5(11.3)
Agree	157(23.6)	38.2%	61.9(12.9)
Strongly agree	46(6.9)	50.0%	59.2(14.3)

**Difficulty accessing health care**			

Strongly disagree	64(9.6)	10.9%	69.1(12.0)
Disagree	261(39.3)	24.9%	66.0(11.6)
Neither agree nor disagree	165(24.9)	30.3%	63.0(11.8)
Agree	144(21.7)	38.9%	58.9(10.3)
Strongly agree	30(4.5)	53.3%	55.0(14.0)

Values are presented as number (%) unless otherwise indicated. SOC values are presented as mean (standard deviation). Some response categories included a small number of participants; therefore, the estimates for these subgroups should be cautiously interpreted.

Table 3 presents the results of the multivariable logistic regression analyses that examined the associations between each COVID-19-related anxiety item and psychological distress (K6 ≥ 5) after adjusting for potential confounding factors. Each anxiety item was entered separately into the regression models together with SOC and covariates. The participants who reported higher fear of infection had increased odds for psychological distress (OR = 1.32, 95% CI: 1.05–1.68). Stress about mask-wearing (OR = 1.35, 95% CI: 1.10–1.68) and concern about reduced income (OR = 1.32, 95% CI: 1.08–1.61) were also significantly associated with psychological distress. Other anxiety items, including perceived discrimination, restricted leisure activities, restricted occasions to meet family or friends, difficulty dining out, and difficulty accessing healthcare, were not significantly associated with psychological distress. Across all models, the SOC (per standard deviation increase) showed inverse associations with psychological distress, with nearly identical point estimates.

**Table 3:** Associations of COVID-19-related anxiety and SOC with psychological distress (K6 ≥ 5)

**Variable**	**OR**	**95% CI**	**p-value**
Fear of infection	1.32	1.05–1.68	0.021
SOC (per SD increase)	0.25	0.19–0.33	<0.001
Fear of discrimination if infected	1.15	0.93–1.43	0.192
SOC (per SD increase)	0.24	0.18–0.32	<0.001
Stress about mask-wearing	1.35	1.10–1.68	0.005
SOC (per SD increase)	0.25	0.18–0.32	<0.001
Restricted leisure or travel	1.13	0.93–1.37	0.226
SOC (per SD increase)	0.24	0.18–0.32	<0.001
Inability to meet family or friends	1.08	0.90–1.31	0.399
SOC (per SD increase)	0.24	0.18–0.32	<0.001
Difficulty dining out	1.01	0.83–1.22	0.934
SOC (per SD increase)	0.24	0.18–0.31	<0.001
Concern about reduced income	1.32	1.08–1.61	0.008
SOC (per SD increase)	0.25	0.18–0.33	<0.001
Difficulty accessing healthcare	1.07	0.88–1.30	0.502
SOC (per SD increase)	0.24	0.18–0.32	<0.001

Results from multivariable logistic regression models examining the association between COVID-19-related anxiety factors and psychological distress (K6 ≥ 5) among all participants (n = 664). Each anxiety item was assessed on a five-point Likert scale (1–5) and treated as a continuous variable in the analyses. Each anxiety factor was entered separately into the model together with sense of coherence (SOC) and covariates. Models were adjusted for age, sex, subjective health status, and communicative health literacy.

Lifestyle variables (smoking status, alcohol consumption, sleep condition, and exercise habit) were not included in these models because they were assessed only among participants aged <75 years in the health check-up questionnaire.

SOC was modeled per one standard deviation (SD) increase to facilitate interpretation.

OR, odds ratio; CI, confidence interval; SOC, sense of coherence.

In a sensitivity analysis restricted to participants aged <75 years with additional adjustment for lifestyle factors (smoking status, alcohol consumption, sleep condition, and exercise habit), the results were largely unchanged, and the magnitude and direction of the associations remained consistent with the primary analyses (Table 4).

**Table 4:** Associations of COVID-19-related anxiety and SOC with psychological distress (K6 ≥ 5) among participants aged <75 years

**Variable**	**OR**	**95% CI**	**p-value**
Fear of infection	1.32	1.02–1.71	0.034
SOC (per SD increase)	0.21	0.15–0.29	<0.001
Fear of discrimination if infected	1.06	0.84–1.35	0.610
SOC (per SD increase)	0.21	0.15–0.28	<0.001
Stress about mask-wearing	1.40	1.12–1.77	0.004
SOC (per SD increase)	0.21	0.15–0.29	<0.001
Restricted leisure or travel	1.16	0.94–1.34	0.156
SOC (per SD increase)	0.21	0.15–0.28	<0.001
Inability to meet family or friends	1.05	0.86–1.29	0.613
SOC (per SD increase)	0.21	0.15–0.28	<0.001
Difficulty dining out	0.98	0.79–1.18	0.749
SOC (per SD increase)	0.20	0.14–0.28	<0.001
Concern about reduced income	1.33	1.07–1.67	0.010
SOC (per SD increase)	0.21	0.15–0.29	<0.001
Difficulty accessing healthcare	1.01	0.82–1.25	0.920
SOC (per SD increase)	0.21	0.15–0.28	<0.001

Results from multivariable logistic regression models examining the association between COVID-19-related anxiety factors and psychological distress (K6 ≥ 5) among participants aged <75 years (n = 590). Each anxiety factor was entered separately into the model together with sense of coherence (SOC) and covariates. Models were adjusted for age, sex, subjective health status, communicative health literacy, smoking status, alcohol consumption, sleep condition, and exercise habit.

SOC was modeled per one standard deviation (SD) increase to facilitate interpretation.

OR, odds ratio; CI, confidence interval; SOC, sense of coherence.

## Discussion

In this study, we examined the associations between COVID-19-related anxiety and psychological distress among rural residents in Japan during the later phase of the pandemic. We found that anxiety related to fear of infection, mask use, and reduced income was associated with higher odds of psychological distress, while a higher sense of coherence (SOC) was associated with lower psychological distress.

The psychological impact of COVID-19 has mostly been assessed in the earlier phase. In a systematic review, measures such as mandatory quarantine, stay-at-home orders, restriction of socioeconomic activities, and uncertainty due to a lack of information were reported as sources of psychological distress [7]. In rural regions in Japan, including Shimane, the COVID-19 incidence remained low during the early pandemic waves and increased substantially starting in early 2022 [33]. This shift may have resulted in the higher levels of infection-related anxiety in our study. After the transition to later pandemic waves, COVID-19 may have affected the participants’ mental health in different ways than in the earlier waves.

The period defined as the late phase in this study (July 2022–March 2023) corresponded to the seventh and eighth waves of COVID-19 in Japan, during which the number of reported infections reached the highest levels since the start of the pandemic [34]. Simultaneously, behavioral restrictions had been largely relaxed, and social and economic activities were gradually returning to normal. This combination of high infection risk in terms of case numbers and normalized daily life created a distinctive psychological environment. Such an ambivalent context may have influenced residents’ perceptions of infection risk and contributed to persistent anxiety despite the easing of public health restrictions.

Prior studies have reported that concerns about stigma, negative evaluations, and community rumors were important contributors to psychological distress during the COVID-19 pandemic [35, 36]. However, in the present study, perceived discrimination and stigma-related anxiety were not associated with psychological distress. One possible explanation is that changes in public awareness or adaptation to the pandemic in the late phase may have attenuated the psychological impact of stigma over time. Nevertheless, in rural settings where social networks are close and privacy is limited, concerns related to social evaluation may indirectly influence mental health by amplifying infection-related fears.

International studies have demonstrated that rural residents often have a lower adherence to preventive behaviors, including mask wearing [21, 37]. In Japan, mask use was widely accepted in Japan even before the COVID-19 pandemic, mask-wearing has long been socially normalized and widely practiced in daily life [38]. Mask-wearing-related stress was significantly associated with psychological distress in our study. Prolonged mandatory use, discomfort, and high social expectations may have resulted in psychological fatigue.

In addition, mask wearing in Japan is strongly influenced by social norms and concerns about social evaluation, which may create pressure to conform to preventive behaviors. Previous studies have suggested that individuals may wear masks not only for infection prevention but also to avoid negative evaluation from others [39]. Such socio-cultural expectations may have amplified mask-related stress during the pandemic. The combination of high compliance and accumulated psychological burden may represent a specific pattern that differs from other rural populations.

Our results were consistent with evidence from several countries showing that economic insecurity significantly worsened mental health during the pandemic [35, 36]. Although official data recorded a modest increase in average gross agricultural income in 2020, the COVID-19 shock coincided with structural challenges such as a significant reduction in the number of agricultural management entities reported in the most recent agricultural census, indicating heightened economic vulnerability among farm households [40, 41]. In addition, international evidence from the Asia-Pacific region suggests that many agricultural and fishing workers experienced income losses during the pandemic, indicating that income instability was a widespread concern [42]. These economic pressures may have amplified psychological distress related to income reduction in rural agriculture-dependent communities. However, because participants in this study were recruited from individuals attending JA health check-ups, the study population may represent farmers with relatively higher health consciousness and stronger engagement with organized services. Consequently, economically vulnerable individuals who do not participate in regular health check-ups may have been underrepresented. In this case, the observed association between concern about reduced income and psychological distress may represent a conservative estimate.

Importantly, SOC was inversely associated with psychological distress in our study. Individuals with higher SOC were less likely to report psychological distress even when experiencing elevated levels of pandemic-related anxiety. These findings align with Antonovsky’s salutogenic framework [10] and previous studies showing that SOC enhances mental resilience during stressful circumstances, including the COVID-19 pandemic [12, 15, 37]. Therefore, individuals with higher SOC may be better able to interpret pandemic-related stressors and mobilize coping resources, buffering the psychological impact of prolonged uncertainty. However, because this study employed a cross-sectional design, causal direction cannot be determined. Individuals experiencing higher psychological distress levels may temporarily perceive their life circumstances as less comprehensible or manageable, which could result in lower reported SOC levels. Therefore, the observed association should be interpreted cautiously; longitudinal studies are needed to clarify the directionality of the relationship between SOC and psychological distress.

From a public health perspective, these findings underscore the need to address multidimensional stressors, including infection-related fear, accumulated psychological fatigue due to prolonged behavioral restrictions, and financial insecurity, particularly in rural regions with an aging population. Strengthening SOC may serve as a strategy for enhancing community resilience. Interventions such as stress management training, improving social connectedness, strengthening community support structures, and providing clear and inclusive health communication have been shown to enhance SOC [12]. Integrating these approaches into local health systems may improve preparedness for future public health emergencies.

This study has some limitations. First, due to its cross-sectional design, causal relationships between pandemic-related anxiety and psychological distress cannot be inferred. Second, our study was subject to selection bias because the participants were recruited from those in a health checkup program; thus, they probably had a higher level of health consciousness. In addition, because information on individuals who did not consent to participate was not available, we were unable to assess whether non-consent occurred randomly. Third, all measures, including COVID-19-related anxiety, K6, and SOC, were assessed using self-administered questionnaires, which may have introduced reporting biases, such as those due to social desirability. In addition, because the questionnaires were completed at home prior to the health check-ups, the possibility that responses may have been influenced by family members cannot be completely excluded. Fourth, we controlled for potential confounding factors in the logistic regression analyses; however, there remains the possibility that unmeasured confounding factors altered the observed associations. However, in a sensitivity analysis restricted to participants aged <75 years with additional adjustment for lifestyle factors (smoking status, alcohol consumption, sleep conditions, and exercise habits), the results remained materially unchanged, suggesting that residual confounding by lifestyle factors is unlikely to explain the observed associations. Underlying mental disorders may have affected the results. Other potentially unmeasured confounding factors include household socioeconomic status and social support; however, these data were not collected. Furthermore, participants excluded due to missing questionnaire responses were slightly older than those included in the final analysis, which may have introduced some degree of selection bias. Fifth, because the participants were members of a specific cooperative health program with a large proportion engaged in agriculture in Shimane Prefecture, the findings may not be fully generalizable to other rural communities in Japan. Rural areas in Japan vary substantially in terms of social structure, economic activities, and population characteristics. Therefore, caution is warranted when extrapolating these findings to rural populations with different occupational compositions or socioeconomic conditions. Finally, although the study period was in the late phase of the pandemic, it was one of the advantages; the psychological distress in the earlier phase was unknown. Future research should include more diverse populations and employ longitudinal designs to examine the temporal changes in pandemic-related anxiety, psychological distress, and SOC.

## Conclusions

In this community-based study conducted during the late phase of the COVID-19 pandemic in a rural region of Japan, several pandemic-related anxiety factors were associated with psychological distress: fear of infection, mask-wearing stress, and concerns about reduced income. SOC was inversely associated with psychological distress, suggesting its potential protective role during a prolonged pandemic. These findings highlight the importance of addressing persistent stressors and strengthening community resilience in rural settings.

## Abbreviations

CI: confidence interval
COVID-19: coronavirus disease 2019
JA: Japan Agricultural Cooperatives
K6: Kessler 6-item Psychological Distress Scale
OR: odds ratio
SOC: sense of coherence
SOC-13: Sense of Coherence 13-item scale

## Declarations

### Availability of data and materials

The datasets generated and analyzed during the current study are not publicly available due to privacy and ethical restrictions; however, they are available from the corresponding author upon reasonable request.

## References

[1] World Health Organization. Public health and social measures (PHSMs) in response to COVID-19 [Internet]; 2020. Geneva: WHO. Accessed 2025 Oct 10. https://www.who.int/emergencies/diseases/novel-coronavirus-2019/phsm.

[2] World Health Organization. Coronavirus disease (COVID-19): advice for the public [Internet]; 2020. Geneva: WHO. Accessed 2025 Oct 10. https://www.who.int/emergencies/diseases/novel-coronavirus-2019/advice-for-public.

[3] Hale T, Angrist N, Goldszmidt R, Kira B, Petherick A, Phillips T, . A global panel database of pandemic policies (Oxford COVID-19 government response tracker). Nat Hum Behav. 2021;5:529–38. doi: 10.1038/s41562-021-01079-8. 33686204

[4] Cabinet Secretariat, Government of Japan. Basic policies for novel coronavirus disease control (COVID-19) [Internet]; 2020–2022. Tokyo: Government of Japan. Accessed 2025 Oct 10. https://www.cas.go.jp/jp/influenza/novel_coronavirus.html.

[5] Ministry of Health, Labour and Welfare. Situation report on COVID-19; 2022. Japan [Internet]. In: Tokyo: MHLW. Accessed 2025 Oct 10. https://www.mhlw.go.jp/stf/seisakunitsuite/bunya/newpage_00032.html.

[6] Brooks SK, Webster RK, Smith LE, Woodland L, Wessely S, Greenberg N, . The psychological impact of quarantine and how to reduce it: rapid review of the evidence. Lancet. 2020;395:912–20. doi: 10.1016/S0140-6736(20)30460-8. 32112714 PMC7158942

[7] Xiong J, Lipsitz O, Nasri F, Lui LMW, Gill H, Phan L, . Impact of COVID-19 pandemic on mental health in the general population: a systematic review. J Affect Disord. 2020;277:55–64. doi: 10.1016/j.jad.2020.08.001. 32799105 PMC7413844

[8] Cohen S, Janicki-Deverts D, Miller GE. Psychological stress and disease. JAMA. 2007;298:1685–7. doi: 10.1001/jama.298.14.1685. 17925521

[9] Sapolsky RM. Why zebras don’t get ulcers. 3rd ed. New York: Holt Paperbacks; 2004.

[10] Antonovsky A. Unraveling the mystery of health: how people manage stress and stay well. San Francisco: Jossey-Bass; 1987.

[11] Kelner M. Antonovsky’s sense of coherence scale and its relation to mental health. Can J Psychiatry. 1988;33:174–9.

[12] Eriksson M, Lindström B. Antonovsky’s sense of coherence scale and its relation with quality of life: a systematic review. J Epidemiol Community Health. 2007;61:938–44. doi: 10.1136/jech.2006.056028. 17933950 PMC2465600

[13] Kwon S. Coping behaviors and mental health during the COVID-19 pandemic: situational and habitual effects on mental distress with hybrid model analysis. Discov Ment Health. 2025;5:114. doi: 10.1007/s44192-025-00263-w. 40751865 PMC12317949

[14] Généreux M, Schluter PJ, Hung KK, Wong CS, Pui Yin Mok C, O’Sullivan T, . One virus, four continents, eight countries: an interdisciplinary and international study on the psychosocial impacts of the COVID-19 pandemic among adults. Int J Environ Res Public Health. 2020;17:8390. doi: 10.3390/ijerph17228390. 33202706 PMC7697775

[15] Schäfer SK, Sopp MR, Schanz CG, Staginnus M, Göritz AS, Michael T. Impact of COVID-19 on public mental health and the buffering effect of a sense of coherence. Psychother Psychosom. 2020;89:386–92. doi: 10.1159/000510752. 32810855 PMC7490493

[16] Tsuno YS, Yamazaki Y. A comparative study of sense of coherence (SOC) and related psychosocial factors among urban versus rural residents in Japan. Pers Individ Dif. 2007;43:449–61. doi: 10.1016/j.paid.2006.12.014.

[17] Pappa S, Ntella V, Giannakas T, Giannakoulis VG, Papoutsi E, Katsaounou P. Prevalence of depression, anxiety, and insomnia among healthcare workers during the COVID-19 pandemic: a systematic review and meta-analysis. Brain Behav Immun. 2020;88:901–7. doi: 10.1016/j.bbi.2020.05.026. 32437915 PMC7206431

[18] Matsuo T, Kobayashi D, Taki F, Sakamoto F, Uehara Y, Mori N, . Prevalence of health care worker burnout during the coronavirus disease 2019 (COVID-19) pandemic in Japan. JAMA Netw Open. 2020;3:e2017271. doi: 10.1001/jamanetworkopen.2020.17271. 32749466 PMC7403916

[19] Huckins JF, daSilva AW, Wang W, Hedlund E, Rogers C, Nepal SK, . Mental health and behavior of college students during the early phases of the COVID-19 pandemic: longitudinal smartphone and ecological momentary assessment study. J Med Internet Res. 2020;22:e20185. doi: 10.2196/20185. 32519963 PMC7301687

[20] Son C, Hegde S, Smith A, Wang X, Sasangohar F. Effects of COVID-19 on college students’ mental health in the United States: interview survey study. J Med Internet Res. 2020;22:e21279. doi: 10.2196/21279. 32805704 PMC7473764

[21] Xiao Y, Becerik-Gerber B, Lucas G, Roll SC. Impacts of working from home during COVID-19 pandemic on physical and mental well-being of office workstation users. J Occup Environ Med. 2021;63:181–90. doi: 10.1097/JOM.0000000000002097. 33234875 PMC7934324

[22] Shimane Prefectural Government. Area and population of Shimane Prefecture [Internet]. Shimane Statistical Information Database; 2024. Accessed 2025 Jul 30. https://pref.shimane-toukei.jp/index.php?view=3830.

[23] Statistics Bureau of Japan. Labour force by industry (Shimane Prefecture) [Internet]. e-Stat; 2023. Accessed 2025 Jul 30. https://www.e-stat.go.jp/.

[24] Japan Institute for Labour Policy and Training. Number of employed persons by industry: labour force survey time-series data [Internet]; 2023. Tokyo: JILPT. Accessed 2025 Jul 30. https://www.jil.go.jp/kokunai/statistics/timeseries/html/g0204.html.

[25] Ministry of Health, Labour and Welfare. Survey on mental health related to COVID-19: data book [Internet]; 2021. Tokyo: Ministry of Health, Labour and Welfare. Accessed 2025 Nov 10. https://www.mhlw.go.jp/content/12200000/datasyuu.pdf.

[26] Kessler RC, Andrews G, Colpe LJ, Hiripi E, Mroczek DK, Normand SL, . Short screening scales to monitor population prevalences and trends in non-specific psychological distress. Psychol Med. 2002;32:959–76. doi: 10.1017/S0033291702006074. 12214795

[27] Furukawa TA, Kawakami N, Saitoh M, Ono Y, Nakane Y, Nakamura Y, . The performance of the Japanese version of the K6 and K10 in the World Mental Health Survey Japan. Int J Methods Psychiatr Res. 2008;17:152–8. doi: 10.1002/mpr.257. 18763695 PMC6878390

[28] Prochaska JJ, Sung HY, Max W, Shi Y, Ong M. Validity study of the K6 scale as a measure of moderate mental distress based on mental health treatment need and utilization. Int J Methods Psychiatr Res. 2012;21(2):88–97. doi: 10.1002/mpr.1349. 22351472 PMC3370145

[29] Togari T, Yamazaki Y. Examination of the reliability and factor validity of the 13-item five-point version sense of coherence scale. Jpn J Health Hum Ecol. 2005;71:168–82. doi: 10.3861/jshhe.71.168.

[30] Idler EL, Benyamini Y. Self-rated health and mortality: a review of twenty-seven community studies. J Health Soc Behav. 1997;38:21–37. doi: 10.2307/2955359. 9097506

[31] Ishikawa H, Kiuchi T. Health literacy and health communication. Biopsychosoc Med. 2010;4:18. doi: 10.1186/1751-0759-4-18. 21054840 PMC2990724

[32] Ishikawa H, Nomura K, Sato M, Yano E. Developing a measure of communicative and critical health literacy: a pilot study of Japanese office workers. Health Promot Int. 2008;23:269–74. doi: 10.1093/heapro/dan017. 18515303

[33] Shimane Prefectural Government. COVID-19 situation updates in Shimane Prefecture; 2022. Shimane, Japan. https://www1.pref.shimane.lg.jp/contents/kansen/topics/2019nCoV/graph.html. Accessed 2025 Dec 22.

[34] Ministry of Health, Labour and Welfare. Visualizing the data: information on COVID-19 infections in Japan [Internet]. Tokyo: Ministry of Health, Labour and Welfare; 2023. Available from: https://covid19.mhlw.go.jp/. Accessed 2026 Mar 17.

[35] He H, Zhou Y, Li M, Shi L. Stigma and discrimination during the COVID-19 pandemic: a global phenomenon. Curr Psychol. 2021;40:6283–94.

[36] Sawamoto R, Goto R, Hayashi Y, Ikezaki S, Hirano H. Stigma toward healthcare workers during the COVID-19 pandemic. Int J Environ Res Public Health. 2023;20:2612.36767978

[37] Callaghan T, Lueck JA, Trujillo KL, Ferdinand AO. Rural and urban differences in COVID-19 prevention behaviors. J Rural Health. 2021;37:287–95. doi: 10.1111/jrh.12556. 33619836 PMC8013340

[38] Li T, Yamaguchi H, Sato T, . Habitual mask wearing as part of COVID-19 control in Japan. Behav Sci (Basel). 2023;13(11):951. doi: 10.3390/bs13110951. 37998697 PMC10669277

[39] Nakayachi K, Ozaki T, Shibata Y, Yokoi R. Why do Japanese people use masks against COVID-19, even though masks are unlikely to offer protection from infection? Front Psychol. 2020;11:1918. doi: 10.3389/fpsyg.2020.01918. 32849127 PMC7417658

[40] Statistics Bureau of Japan. Statistical handbook of Japan 2022; 2022. Tokyo: Statistics Bureau, Ministry of Internal Affairs and Communications. https://www.stat.go.jp/english/data/handbook/pdf/2022all.pdf.

[41] Ministry of Agriculture, Forestry and Fisheries (MAFF). 2025 Census of Agriculture and Forestry: Preliminary Results; 2025. Tokyo: MAFF. https://www.maff.go.jp/j/tokei/census/afc/index.html.

[42] Food and Agriculture Organization of the United Nations. The Impact of COVID-19 on Fisheries and Aquaculture Food Systems, Possible Responses and Coping Strategies in the Asia-Pacific Region; 2021. Rome: FAO. https://openknowledge.fao.org/.

